# Japanese Consumer Perceptions of Genetically Modified Food: Findings From an International Comparative Study

**DOI:** 10.2196/ijmr.5850

**Published:** 2016-08-29

**Authors:** Keiko Komoto, Sawako Okamoto, Miki Hamada, Naoya Obana, Mami Samori, Tomoaki Imamura

**Affiliations:** ^1^ Nara Medical University Department of Public Health, Health Management and Policy Nara Japan; ^2^ Mitsubishi Research Institute, Inc. Social and Public Management Research Division Tokyo Japan

**Keywords:** genetically modified food, Japan, perception, health, risk

## Abstract

**Background:**

Reports of food-related incidents, such as cows infected with bovine spongiform encephalopathy (2001) and the Fukushima nuclear accident (2011), engendered significant fear among Japanese consumers and led to multiple farmer suicides, even when no actual health damage occurred. The growing availability of genetically modified (GM) food is occurring against this backdrop of concern about food safety. Consumers need information to assess risk and make informed purchasing decisions. However, we lack a clear picture of Japanese consumer perceptions of GM food.

**Objective:**

This study aims to understand Japanese consumer perceptions of GM food for risk communication. Consumer perceptions of GM food were compared among 4 nations.

**Methods:**

A Web-based survey was conducted in Japan, the United States, the United Kingdom, and France. Participants were asked about demographics, fear of health hazards, resistance to GM and breeding-improved products, perception of GM technology and products, and willingness to pay. Multiple linear regression analyses were conducted, as were *t* tests on dichotomous variables, and 1-way analysis of variance and post hoc tests.

**Results:**

Of 1812 individuals who agreed to participate, 1705 (94%) responded: 457 from Japan and 416 each from France, the United States, and the United Kingdom. The male/female and age group ratios were all about even. Some resistance to GM food was seen in all countries in this study. France showed the strongest resistance (*P*<.001), followed by Japan, which had stronger resistance than the United States and the United Kingdom (*P*<.001). Overall, females, people in their 60s and older, and those without higher education showed the greatest resistance to GM food. Japan showed stronger fear of food hazards than other nations *(P*<.001, odds ratio=2.408, CI: 1.614-3.594); Japanese and French respondents showed the strongest fear of hazards from GM food (*P*<.001). Regarding perceptions of GM technology and products, consumers in nations other than Japan would accept GM food if it were appropriately explained, they were provided with scientific data supporting its safety, and they understood that all food carries some risk. However, Japanese consumers tended to accept GM technology but rejected its application to food (*P*<.001). Of those willing to purchase GM food, consumers in Japan required a discount of 30% compared with about 20% in other nations.

**Conclusion:**

All consumers in our study showed resistance to GM food. Although no health hazards are known, respondents in Japan and France strongly recognized GM food as a health risk. Price discounts of 30% and GM technology may be communication cues to start discussions about GM food among Japanese consumers. Although education-only risk communication generally is not effective, such an approach may work in Japan to help consumers better understand GM technology and, eventually, GM food. The gap between accepting GM technology and rejecting its application to food should be explored further.

## Introduction

### Background

Advances in recombinant DNA technology have led to the growing worldwide availability of genetically modified (GM) food. However, consumer acceptance of GM food in Japan and in Western countries remains low. Previous studies reveal the following concerns among consumers about the possible effects of GM food: health hazards from consuming GM food, including long-term effects, negative ecological impacts, effects on future generations, and limited purchasing options that may result from uncontrolled dominance of GM food [1-5]. On the other hand, consumers also consider the possible advantages of GM food, such as helping to keep down the overall cost of food, reduced waste, and longer shelf life [1,4]. In addition, some studies indicate that trust and other emotions influence perceptions of GM food [6-8]. Under these circumstances, it is important to know consumers’ perceptions and acceptance of GM food so that risk communication can effectively influence purchasing behaviors and help consumers make well-informed decisions [9-13].

In Japan, Tanaka [14] reported that Japanese people have negative feelings or attitudes toward GM food. Imamura et al [5] have led a series of government-funded studies of the public acceptance of GM food since 2009. s in studies of GM food in Western developed nations, the researchers used mass media trends as an index of public acceptance, held a focus group with consumers who have resistance to GM products overall, and conducted a Web-based survey on GM products that included a comparison between GM food and other food. Their results indicate that about 70% of the Japanese consumers surveyed did not want to eat GM food and had lower acceptance of GM food compared with food containing natural toxins and additives. Contrary to the reports by Tanaka [14] and Imamura et al [5], some studies reported that GM food was more accepted in Japan and the United States than in European countries [2,15].

Furthermore, it seems that consumer resistance to GM food may stem from uncertainty and/or unwarranted concern associated with GM technology and its use in the production of GM food [16-19]. People usually seek to reduce their uncertainty by gathering information and trying to better understand the issue of concern [20]. However, it seems that Japanese consumers do not demonstrate such attitudes [5] and a different explanation may apply. In the case of Japanese consumers, when unexpected information is given, that information may actually increase rather than decrease their uncertainty [21]. Or, they may create certainty by making up their minds about issues/products based solely on certain confirmed information and may be reluctant to communicate any remaining uncertainties [9,22]. Furthermore, when information is too complicated to understand, Japanese people tend to think based on their preexisting attitudes rather than on newly acquired information [23].

From a cultural perspective, uncertainty can be viewed as a cultural trait in response to risk and ambiguity [24]. People with low levels of tolerance for ambiguity have high levels of uncertainty avoidance and a desire for clear answers and solutions. In contrast, people with high tolerance for ambiguity have low uncertainty avoidance and tend to accept ambiguous answers and shades of gray [25]. One study reports that, even for countries as a whole, high uncertainty avoidance is negatively correlated with risk taking and positively correlated with fear of failure; France and Japan are included in the high uncertainty avoidance group, whereas the United States and the United Kingdom are in the low uncertainty avoidance group. Countries with high uncertainty avoidance tend to display emotion more than countries with low uncertainty avoidance [26]. At the same time, Japan places a high importance on social balance and harmony, is group oriented (collectivism), and discourages verbal communication in formal situations. These cultural traits mean that Japanese people tend to accept verbal ambiguity during communication and generally refrain from expressing personal opinions or attitudes [27,28]. Therefore, we believe that there may be a cultural predisposition that may influence Japanese consumers’ ability or willingness to accept GM foods.

### Focus of This Study

Evidence regarding Japanese consumer attitudes is mixed, making it difficult to understand their perceptions of GM food. Consumer risk perceptions are influenced and created by scientific evidence and experts’ opinions, as well as social, economic, political, and psychological factors [8-13,29,30]. Risk communication that disseminates information without first recognizing the nature of the public’s risk perception is likely to be pointless and ineffective.

This study aims to illustrate the distinctive traits of Japanese consumer perceptions of GM food. Taking into consideration that consumer concerns vary for different kinds of food hazards [31], the authors expected to obtain useful data regarding public perceptions of GM food in comparison with consumers in other nations. The Western nations in our study have each conducted studies of their own consumer perceptions, including some international comparative studies of consumer perceptions of GM food [2,15,19]. Only 2 of these studies included Japan in their comparisons. Furthermore, no international investigations and comparisons to understand Japanese consumer perceptions and attitudes have been done since 2001, when labeling of all GM food on the market became a legal requirement in Japan [32]. The outcomes in this study are therefore useful not only for Japan but also for all countries that participated in this study of consumer perceptions of GM food, as well as other countries with cultures similar to Japan’s.

### Research Framework and Hypotheses Development

In this research, the authors applied the elaboration likelihood model (ELM) [23] of attitude change. In this theory, motivation and ability are both required for someone to evaluate an issue. If the person’s motivation and ability are high, the “elaboration likelihood” is also high that the person will think about the issue in depth. Information that deeply concerns the person is repeatedly processed and stored in long-term memory (central route). Favorable cognitive responses will be elicited only if the message arguments are compelling for the recipients.

If, on the other hand, the message arguments are not compelling (ability and motivation are low), people may need to have the information early or be reminded by communication cues, such as earning potential, attractive information sources, and situational stimuli. If the public does not have much prior information about the perceived issue, or if the issue does not have much personal relevance, it will be necessary for people to constantly be reminded by the previously mentioned communication cues (peripheral route). If this approach is successful, the information is nevertheless likely to be temporary and short lived. However, such a short-term choice/opinion change may create dissonance within the person, who will become motivated to think about a different choice/opinion and create bolstering cognitions that then may lead to a more permanent change in attitude.

A previous study [33] suggested that ELM may be useful to elicit determinants of effective risk communication. Although ELM did not predict attitude change, this theory could nevertheless be used as a tool to understand consumers’ information processing [34]. Taking into consideration the ELM theory and previous study results, this study adopts ELM as a framework from which to examine and understand consumers’ perceptions, including their ideas, beliefs, and ability to take in information as a result of how they understand GM food.

In line with previous studies of resistance to GM organisms [3,15], Tanaka [14] and Imamura et al [5] also found that Japanese consumers may not clearly understand the multiple benefits and risks of GM food. Furthermore, even though Japanese consumers are not fully informed about GM food, they have a strong resistance to it, and the authors speculated they might be particularly concerned about health hazards from GM food that have yet to be identified [5]. Tanaka [14] reported that only demographic factors of gender and age are related to the attitudes of Japanese people toward GM food. Perception and acceptance of GM technology vary depending on its use; in the EU nations and the United States, medical use of GM technology may be more strongly supported than its use in agriculture [35]. Based on this information and using ELM as a framework, we propose the following hypotheses about the perceptions of Japanese people toward GM food: (H1) only age and gender are related to attitudes toward GM food in Japan; (H2) fear of health hazards, as a personal relevance, disturbs the intent to understand GM; (H3) the label breeding-improved product is more acceptable than GM food; (H4) consumers have strong resistance to GM food; (H5) consumers have strong resistance to GM technology; and (H6) consumers do not have the intent to understand GM food.

This study was conducted with the approval of the Ethics Committee of Nara Medical University (authorization code: 655).

## Methods

### Data Collection

This study targeted consumers in 4 nations: Japan, the United States, the United Kingdom, and France. These countries were selected due to their stances on GM food, as shown clearly at the second session of the CODEX ad hoc intergovernmental task force on foods derived from biotechnology in March 2001; the United States was in favor, France was opposed, and the United Kingdom and Japan were neutral (CODEX, ALINORM01/34A).

A Web-based survey was carried out by the Internet research company Macromill Inc and Tokyo from April 20 to May 14, 2013. The company recruited 38,588 people who lived in each country and had registered as monitors; these monitors had participated in various other Web-based marketing research studies and represented diverse regions within each country. This Web-based company distributed the questionnaire through emails to monitors who indicated they would respond. Since the younger 20s generation showed a lower response rate in previous GM-related surveys in Japan [5], the recruiting email and survey questionnaire were distributed only to monitors 30 years and older in this study. To prevent spamming, invalid responses and fake registrations, the company conducts quality control reviews of each monitor’s information once a year.

Participants were stratified into 4 age groups: 30s, 40s, 50s, and 60s and older in each country. For the duration of the study, the company distributed the questionnaire to arrive at the target number of 400-450 in each age group.

The questionnaire was developed by the authors and administered after conducting a pilot study, applying exploratory factor analysis (EFA) and confirmatory factor analysis (CFA); this questionnaire has been used several times in other research in Japan [5]. Although this study focused on GM food, we intentionally included items in the questionnaire about other GM products as filler items to avoid boredom and automatic responses. “Consumers” in this paper means people who are not experts in GM technology and GM food; experts were excluded when recruiting participants. The questionnaire included general questions, such as “do you usually mind seeing GM products when you go grocery shopping?” to learn about respondents’ basic perceptions of GM food.

After collecting the questionnaire, EFA and CFA were conducted to ensure that the factor division was appropriate. For the statistical analysis, multiple linear regression analyses were exploratively conducted, and *t* tests were conducted on dichotomous variables. One-way analysis of variance (ANOVA) and post hoc tests (Tukey) were conducted when appropriate. The effect size was examined and the CI was set at 95%. How fear of GM food is related to consumers in the participating countries was examined by chi-square test and odds ratio. SPSS, version 22 and SPSS AMOS version 24 (IBM SPSS Statistics, Tokyo, Japan) were used for data analyses.

Questions were grouped in 7 main categories based on EFA and CFA: (1) demographics, (2) fear of health hazards, (3) resistance to GM products, (4) resistance to breeding-improved products, (5) interest in scientific explanations regarding GM technology and products, (6) intention to understand GM technology and food, and (7) willingness to pay (WTP).

### Fear of Health Hazards

This category consisted of questions directly related to self in terms of ELM’s description of perceived relevance. We included factors that actually pose health risks, such as food poisoning, norovirus, radioactive materials, bovine spongiform encephalopathy (BSE), trans fatty acids, dioxin, acrylamide, and methylmercury. GM food was on this list even though GM food has not been documented as a risk to health. The degree of fear was rated using a 6-point Likert Scale, from 1 “not afraid at all” to 6 “very afraid.”

### Resistance to GM Products

This category was indirectly related to self, according to ELM’s perceived relevance because in these cases consumers can exercise choice. The following 10 GM products were listed: GM salmon that grows twice as fast as traditional salmon; GM shining killifish, whose bodies shine like tropical fish; GM blue roses; GM hay fever-alleviating rice, which reduces symptoms with continuous consumption; GM herbicide-tolerant crops; GM pest-resistant crops; GM nutrient-enriched crops (vitamin C, and so forth); GM drought-tolerant crops; GM cold weather-tolerant crops; and GM rapid-grow apples that grow quickly and are picked from trees earlier than regular apples. The participants were asked to rate their degree of resistance using a 6-point Likert Scale, from 1 “very strong resistance” to 6 “no resistance at all.”

### Resistance to Breeding-Improved Products

This category was created to compare with the resistance to GM products using the same items; the only difference is the use of the term “breeding-improved” instead of “GM,” as in breeding-improved salmon and breeding-improved herbicide-tolerant crops. The participants were asked to rate their degree of resistance using a 6-point Likert Scale, from 1“very strong resistance” to 6 “no resistance at all.”

### Interest in Scientific Explanation of Genetically Modified Technology and Products

To understand Japanese consumers’ interest in scientific explanations regarding GM technology and products, this category included statements like “most consumers would accept GM food if provided with scientific data supporting its safety.” The degree of agreement or disagreement was measured using a 6-point Likert Scale, from 1 “strongly agree” to 6 “strongly disagree.”

### Intention to Understand Genetically Modified Technology and Food

This category was used to identify consumers’ intention to understand GM technology and GM food by eliciting agreement/disagreement with statements, such as “most consumers are not aware of risks to food safety,” “If provided with an explanation of genetically modified technology, most consumers would accept GM food,” and “It is annoying to repeatedly hear the same argument about the safety of GM food.” These questions were included to determine whether emotion and social frame influence Japanese consumers’ perception and attitudes [26]. The degree of agreement or disagreement was measured using a 6-point Likert Scale, from 1 “strongly agree” to 6 “strongly disagree.”

### Willingness to Pay to Measure Resistance to Genetically Modified Food

Although we expected that data obtained from the previously mentioned questions would give us meaningful insights, we considered that a different angle of approach, such as WTP, might yield unexpected findings. The following products were listed in the questionnaire: GM canned corn, GM corn flakes, tomato grown with GM-corn fertilizer, GM chicken thighs, chicken thighs grown with GM-corn feed, wine fermented with GM yeast, and GM blue rose. For the WTP questions, the average market-list prices for non-GM products were indicated in the appropriate currency for each of the 4 countries surveyed. In each country, the average market list price was set as 1, and the ratio of WTP was measured and then compared among countries.

## Results

### Data Collection

Reliability of the questionnaire was examined by Cronbach alpha (.880). EFA and CFA were conducted: EFA indicated factors as in (1) fear of health hazards, (2) resistance to GM products, (3) resistance to breeding-improved products, (4) interest in scientific explanations of GM technology and products, (5) intention to understand GM technology and food; CFA showed a high goodness of fit (CFI=.962, GFI=.921, AGFI=.906, RMSEA=.046).

Out of 38,588 recruiting emails distributed, 1812 recipients (approximately 5%) agreed to participate and 1705 (94%) completed questionnaires were collected. The number of responses from each country was as follows: 457 from Japan, 416 from the United States, 416 from the United Kingdom, and 416 from France. The ratio of male-to-female respondents was approximately 1:1 for all participating countries. The percentage of respondents in their 30s, 40s, 50s, and 60s and older was approximately 25% for each age group. Almost all participants had jobs outside the food industry.

Because education systems varied among the participating countries, we categorized educational attainment as received/did not receive a university education. The ratio of respondents with/without a university education was, respectively, 53% to 47% in Japan, 47% to 53% in the United States, 64% to 36% in the United Kingdom, and 63% to 37% in France. The ratio of having children (0-19 years old) was 34%, 27%, 30%, and 34% in Japan, the United States, the United Kingdom, and France, respectively. Median household income was in the range of 6 million yen in Japan, 50 thousand dollars in the United States, 20 thousand pounds in the United Kingdom, and 30 thousand Euros in France. Respondents who answered that he/she does not want to answer accounted for an all-country average of 10% (16%, 6%, 8%, and 10% in Japan, the United States, the United Kingdom, and France, respectively; Table 1). Although the respondents choosing “do not want to answer” are included in the data, a reduction of responses of about 10% might bias the results. Furthermore, converting household income to a single currency, such as US$, would have created biases based on fluctuating exchange rates and different commodity prices in each country. Purchasing-power parity was taken into consideration, but income in different currencies and countries cannot be simply compared because income is influenced not only by amount but also by varying subsidies, such as health insurance, child support, and educational support. Household income was therefore excluded from further data analyses. In the multiple linear regression analyses, having children did not show significance in most questions.

**Table 1:** Demographics of the investigated countries.

Participants and Demographics			Japan	United States of America	the United Kingdom	France	Total
Agreed to participate			n=481	n=448	n=434	n=449	n=1,812
Recruitment rate (%)			24.7	2.6	3.6	6.0	4.7
Valid			n=457	n=416	n=416	n=416	n=416
Response rate (%)			95.0	92.9	95.9	92.7	94.1
Demographics							
	Gender (%)						
		Male	47.0	50.0	50.0	50.0	49.2
		Female	53.0	50.0	50.0	50.0	50.8
	Age (%)						
		30s	21.9	25.0	25.0	25.0	24.2
		40s	26.7	25.0	25.0	25.0	25.5
		50s	26.0	25.0	25.0	25.0	25.3
		60s and older	25.4	25.0	25.0	25.0	25.1
	Household income (%)						
			<1 million yen – 1.1	<$20,000 – 13.0	<£10,000 – 11.5	<€10,000 – 6.3	
			1 million yen level – 3.9	<$30,000 – 11.8	<£20,000 – 21.4	<€10,000 – 15.9	
			2 million yen level – 8.5	<$40,000 – 11.3	<£30,000 – 19.0	<€20,000 – 24.0	
			3 million yen level – 9.2	<$50,000 – 8.9	<£40,000 – 12.7	<€30,000 – 22.4	
			4 million yen level – 11.4	<$60,000 – 9.4	<£50,000 – 12.3	<€40,000 – 13.7	
			5 million yen level – 10.1	<$70,000 – 10.1	<£60,000 – 5.3	<€50,000 – 3.4	
			6 million yen level – 9.8	<$80,000 – 6.3	<£70,000 – 2.6	<€60,000 – 1.4	
			7 million yen level – 7.0	<$90,000 – 2.6	<£80,000 – 2.4	<€70,000 – 1.7	
			8 million yen level – 6.3	<$100,000 – 6.3	<£90,000 – 2.9	<€80,000 – 0.5	
			9 million yen level – 4.2	<$120,000 – 6.0	<£100,000 – 1.0	<€90,000 – 0.5	
			≥10 million yen – 12.7	<$160,000 – 6.0	<£120,000 – 0.7	≥€100,000 – 0.5	
			Not wish to answer – 15.8	<$200,000 – 1.4	<£160,000 – 0.2	Not wish to answer – 9.9	
				≥$200,000 – 1.4	<£200,000 – 0.2		
				Do not wish to answer – 5.5	≥£200,000 – 0.0		
					Do not wish to answer – 7.7		
	Child or children (%)						
		Yes	33.9	26.7	29.8	33.9	31.1
		No	66.1	73.3	70.2	66.1	68.9

When asked if they usually mind the presence of GM products, 61% of respondents in Japan, 46% in the United States, 58% in the United Kingdom, and 72% in France answered that they minded seeing GM products in their daily grocery shopping. Multiple regression analyses showed significance for country (*P*<.001), gender (*P*<.001), age (*P*=.001), and education (*P*=.044). Females minded GM food significantly more than males with a *t* test (*P*=.001). Among countries, one-way ANOVA showed significance (*P*<.001). The Tukey test showed that respondents in France minded significantly more compared with those in the other 3 countries (*P*<.001). Gender and education did not show significance in the Tukey tests.

### Fear of Health Hazards

#### Comparison of Demographics

Multiple linear regression analyses showed significant differences in gender (*P* ≤.001) except dioxin (*P*=.004); age (*P*<.001) except GM food (*P*=.004); and country (*P*<.001) except trans fatty acid (*P*=.012) and GM food (no significance). With *t* tests, females in Japan, the United States, and the United Kingdom felt fear significantly more than males, but there was no difference in France. The *P* values for each health hazard in each country are as follows: in Japan, norovirus (*P*=.042), radioactive materials (*P*=.012), BSE (*P*=.043), trans fatty acids (*P*=.047), acrylamide (*P*<.001), and methylmercury (*P*<.001); in the United States, norovirus (*P*=.048), radioactive materials (*P*=.012), trans fatty acid (*P*=.003), and GM food (*P*=.002); in the United Kingdom, food poisoning (*P*=.002), norovirus (*P*=.001), radioactive materials (*P*<.001), BSE (*P*<.001), trans fatty acids (*P*<.001), dioxin (*P*=.006), acrylamide (*P*<.001), methylmercury (*P*<.001), and GM food (*P*<.001).

With 1-way ANOVA, all questions regarding health hazards showed significance (*P*<.001) except trans fatty acid (*P*=.003). Accordingly, the post hoc tests (Tukey tests) for age groups showed that the 60s and older generation partially felt significantly stronger fear in several items than the 30s generation: in Japan, dioxin (*P*=.024), acrylamide (*P*=.008) and, methylmercury (*P*=.006); in the United States, BSE (*P*=.048) and methylmercury (*P*=.012); in the United Kingdom, norovirus (*P*=.030), dioxin (*P*<.001), acrylamide (*P*=.031), and methylmercury (*P*=.002).

#### Comparison of Countries

In a comparison among countries with ANOVA, significance was shown on each question regarding health hazards (*P*<.001). In a subsequent Tukey test, Japanese respondents felt significantly stronger fear than those in the other 3 countries for all causes of health hazards except GM food (*P*<.001). Respondents in Japan and France had significantly stronger fear of GM food than those in the United States and the United Kingdom (*P*<.001). There was no difference between Japan and France (Table 2). The effect size between Japan and each country on the other 3 countries for each health hazard is shown in Table 2.

Furthermore, the association between perception of GM food as a health hazard and perception of GM technology and food was examined. These factors are significantly associated for the United States and France. In both countries, respondents who think GM food poses a health hazard agreed with “1. Most consumers are not aware of risks to food safety” (US *P*=.021, odds=1.894, CI: 1.099-3.263; France *P*=.001, odds=3.133, CI: 1.657-5.923) and “2. Most consumers do not understand the risk of GM food” (US *P*=.006, odds ratio=2.500, CI: 1.280-4.885; France *P*<.001, odds ratio=3.677, CI: 1.817-7.442).

Respondents in Japan, the United Kingdom, and France who thought that GM food poses a health hazard significantly associated with “3. If provided with an explanation of GM technology, most consumers would accept GM food” (Japan *P*<.001, odds ratio=0.304, CI: 0.198-0.467; US *P*<.001, odds ratio=0.332, CI: 0.219-0.507; France *P*=.001, odds ratio=0.419, CI: 0.247-0.711).

Respondents in all participating countries who thought that GM food poses a health hazard significantly agreed with “4. Most consumers would accept GM food if provided with scientific data supporting its safety” (Japan *P*<.001, odds ratio=0.439, CI: 0.285-0.677; US *P*<.001, odds ratio=0.403, CI: 0.254-0.641; UK *P*<.001, odds ratio=0.356, CI: 0.227-0.554; France *P*<.001, odds ratio=0.361, CI: 0.202-0.646). Respondents in all participating countries who thought that GM food poses a health hazard significantly associated with “5. Most consumers would accept GM food if they understood that all food carries a certain level of risk” (Japan *P*=.001, odds ratio=0.488, CI: 0.325-0.733; US *P*=.007, odds ratio=0.568, CI: 0.376-0.858; UK *P*<.001, odds ratio= 0.358, CI: 0.235-0.543; France *P*=.001, odds ratio=0.440, CI: 0.274-0.708).

Japan showed significant association between perception that GM food poses a health hazard and “6. Most consumers cannot understand GM technology even if it is explained to them” (*P*<.001, odds ratio=2.408, CI: 1.614-3.594). Respondents in the United States significantly associated perception that GM food poses a health hazard and “7. Consumers should try hard to understand scientific information and learn more about the issue” (*P*=.009, odds ratio=2.200, CI: 1.226-3.948). France significantly associated perception that GM food poses a health hazard and “8. It is annoying to hear the same argument about safety of GM food repeated over and over, even when consumers do not understand it” (*P*=.006, odds ratio=1.919, CI: 1.218-3.022).

**Table 2:** Fear of health hazards from food.^a,b^

Health hazard	Values	Japan	United States	the United Kingdom	France	ANOVA^c^ (F value)	*P*
Food poisoning	Mean (SD^d^)	4.98 (1.019)	4.10 (1.402)	4.09 (1.400)	4.09 (1.359)	51.691	<.001
	Effect size:g(CI)		0.73 (0.59 to 0.86)	0.73 (0.59 to 0.87)	0.74 (0.61 to 0.88)		
Norovirus	Mean (SD)	5.06 (0.981)	3.79 (1.482)	4.00 (1.446)	4.08 (1.434)	78.573	<.001
	Effect size:g(CI)		1.02 (0.88 to 1.16)	0.87 (0.73 to 1.01)	0.81 (0.67 to 0.94)		
Radioactive material	Mean (SD)	5.16 (1.047)	3.94 (1.658)	4.07 (1.612)	4.62 (1.481)	63.824	<.001
	Effect size:g(CI)		0.89 (0.75 to 1.03)	0.81 (0.67 to 0.95)	0.43 (0.29 to 0.56)		
BSE^e^	Mean (SD)	4.80 (1.154)	3.39 (1.370)	3.71 (1.513)	4.27 (1.515)	87.215	<.001
	Effect size:g(CI)		1.12 (0.97 to 1.26)	0.81 (0.68 to 0.95)	0.39 (0.26 to 0.53)		
Trans fatty acids	Mean (SD)	4.05 (1.120)	3.45 (1.368)	3.51 (1.289)	3.81 (1.290)	21.351	<.001
	Effect size:g(CI)		0.48 (0.35 to 0.62)	0.45 (0.31 to 0.58)	0.20 (0.07 to 0.33)		
Dioxin	Mean (SD)	4.95 (1.051)	3.62 (1.454)	3.56 (1.437)	4.40 (1.380)	108.179	<.001
	Effect size:g(CI)		1.05 (0.91 to 1.19)	1.12 (0.97 to 1.26)	0.45 (0.32 to 0.59)		
Acrylic amide in processed foods	Mean (SD)	4.36 (1.146)	3.52 (1.417)	3.47 (1.369)	4.04 (1.368)	45.431	<.001
	Effect size:g(CI)		0.66 (0.52 to 0.79)	0.71 (0.57 to 0.85)	0.25 (0.12 to 0.39)		
Methylmercury in fishery products	Mean (SD)	4.95 (1.059)	3.91 (1.450)	3.82 (1.397)	4.07 (1.422)	66.626	<.001
	Effect size:g(CI)		0.83 (0.69 to 0.96)	0.92 (0.78 to 1.06)	0.71 (0.57 to 0.84)		
GM^f^ food	Mean (SD)	4.07 (1.196)	3.52 (1.490)	3.26 (1.429)	4.20 (1.427)	43.882	<.001
	Effect size:g(CI)		0.41 (0.27 to 0.54)	0.62 (0.48 to 0.75)	−0.10 (−0.23 to 0.03)		

^a^Likert Scale: 1=not afraid at all to 6=very afraid.

^b^Mean: average of Likert Scale points.

^c^ANOVA: analysis of variance.

^d^SD: standard deviation.

^e^BSE: bovine spongiform encephalopathy.

^f^GM: genetically modified.

### Resistance to Genetically Modified Products

#### Comparison of Demographics

Multiple linear regression analyses showed that gender was significantly associated with all GM products (*P*<.001), GM herbicide-tolerant crops (*P*=.035), and GM nutrient-enriched crops (*P*=.001), and country with 6 GM products: GM salmon, shining killifish, hay fever-alleviating rice, cold weather-tolerant crops, rapid-grow apples (*P*<.001, respectively), and GM drought-tolerant crops (*P*=.018). With t tests, females showed significantly stronger resistance to GM products in all 4 countries: in Japan, GM salmon (*P*<.001), GM shining killifish (*P*=.011), GM hay fever-alleviating rice (*P*=.009), GM herbicide-tolerant crops (*P*<.001), GM pest-resistant crops (*P*=.004), GM nutrient-enriched crops (*P*=.039), GM drought-tolerant crops (*P*=.006), GM cold weather-tolerant crops (*P*=.002), and GM rapid-grow apples (*P*<.001); in the United States, GM salmon (*P*<.001), GM shining killifish (*P*=.003), GM hay fever-alleviating rice (*P*=.001), GM herbicide-tolerant crops (*P*=.001), GM pest-resistant crops (*P*=.004), GM drought-tolerant crops (*P*=.011), GM cold weather-tolerant crops (*P*=.001), and GM rapid-grow apples (*P*<.001); in the United Kingdom, GM salmon (*P*<.001), GM shining killifish (*P*<.001), GM blue roses (*P*=.021), GM hay fever-alleviating rice (*P*<.001), GM herbicide-tolerant crops (*P*<.001), GM pest-resistant crops (*P*<.001), GM nutrient-enriched crops (*P*=.018), GM drought-tolerant crops (*P*<.001), GM cold weather-tolerant crops (*P*<.001), and GM rapid-grow apples (*P*<.001); and in France, GM hay fever-alleviating rice (*P*=.010), GM pest-resistant crops (*P*=.034), and GM cold weather-tolerant crops (*P*=.020).

#### Comparison of Countries

**Figure 1:**
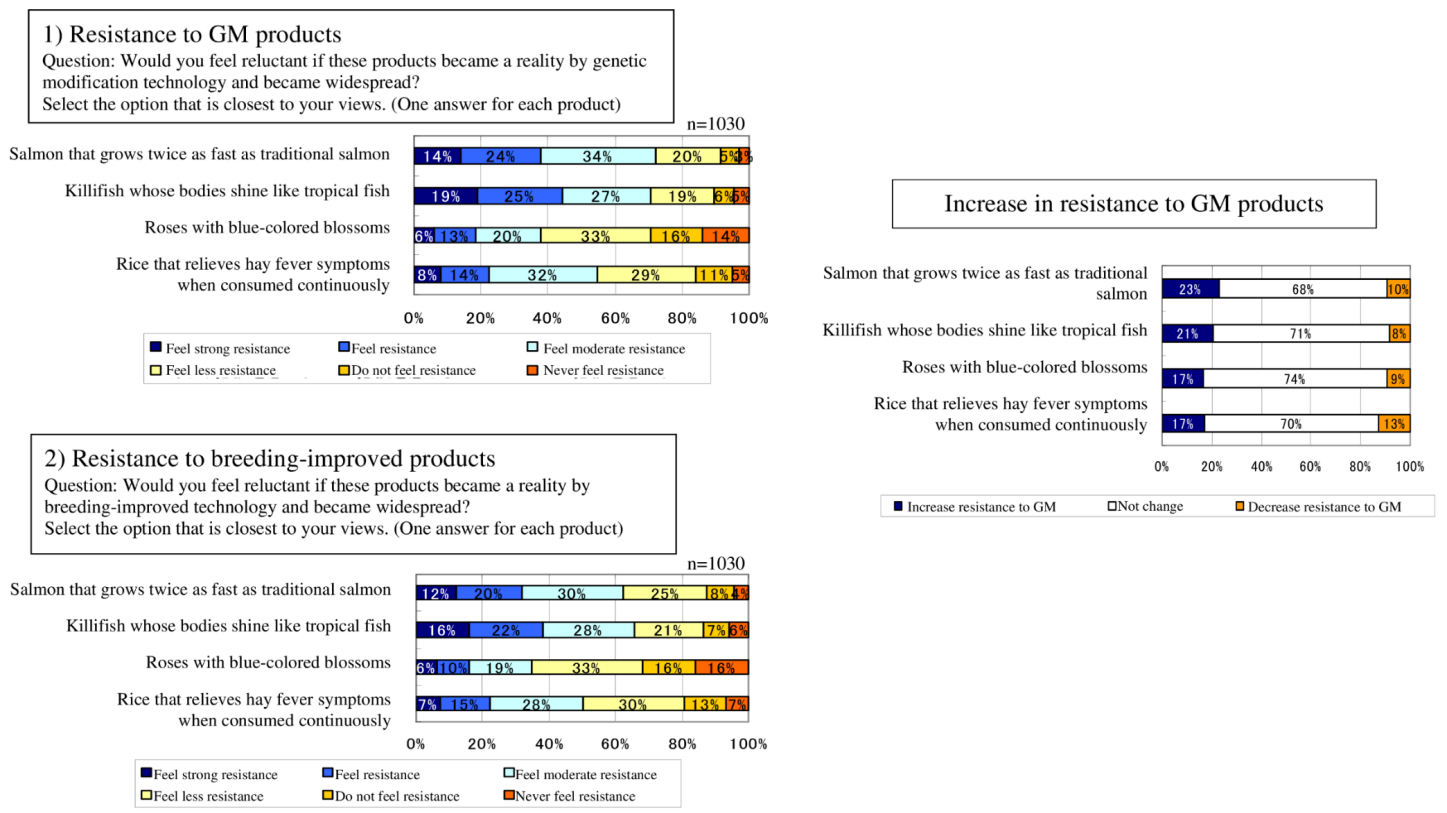
Change in consumer resistance to GM and breeding-improved food. GM: genetically modified.

**Figure 2:**
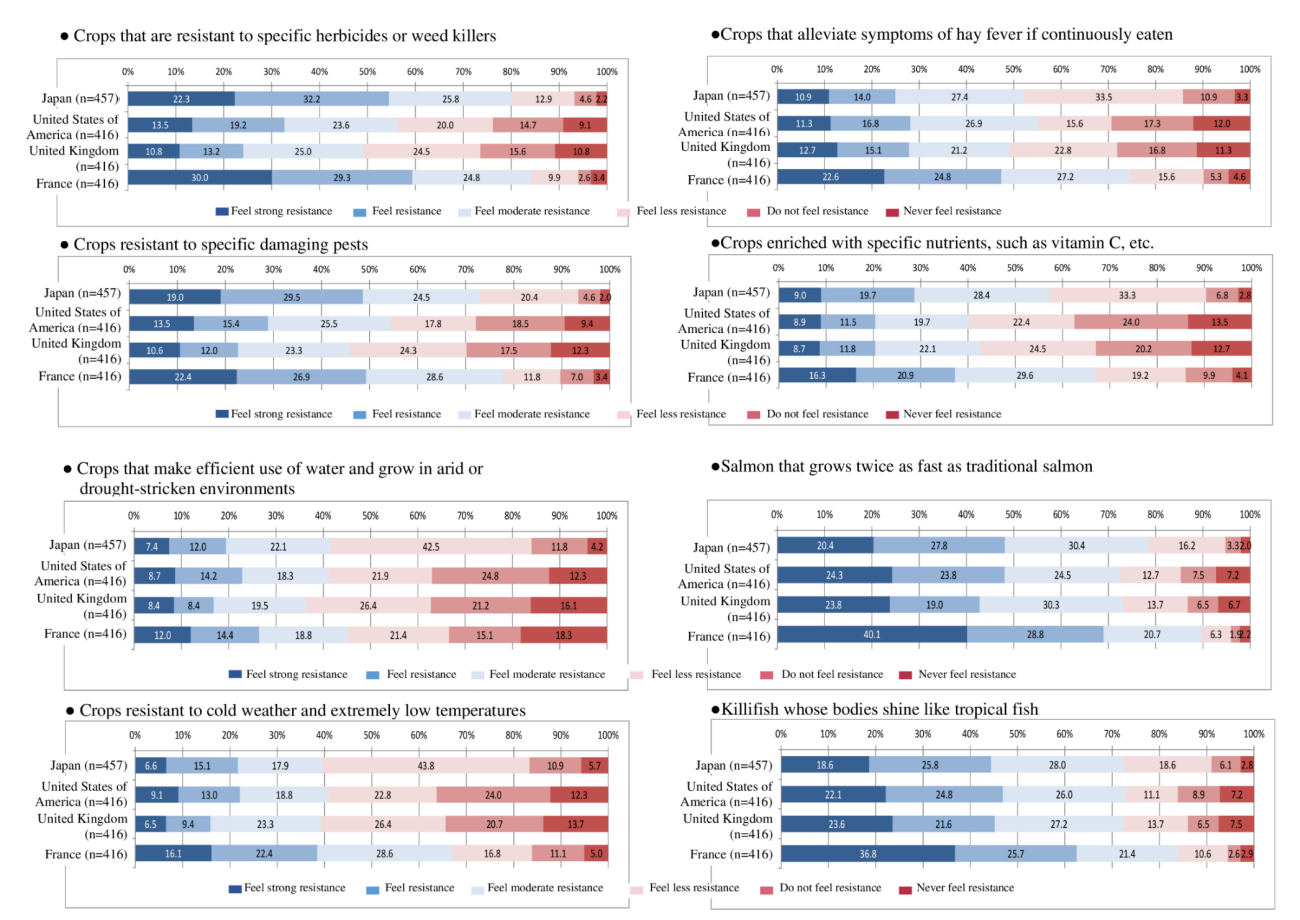
Resistance to GM products. (Respondents were asked to answer only for products available in their country.).

The comparison among countries showed significance in every GM product with 1-way ANOVA (*P*<.001). With Tukey tests, France showed significantly stronger resistance than the other 3 countries for 5 items: GM salmon, GM shining killifish, GM hay fever-alleviating rice, GM cold weather-tolerant crops, and GM rapid-grow apples (*P*<.001, respectively). For GM herbicide-tolerant crops, GM pest-resistant crops, and GM nutrient-enriched crops, respondents in both France and Japan showed significantly stronger resistance than those in the United States and the United Kingdom (Figure 1), with no significant difference between France and Japan (*P*<.001 in Japan and *P*<.001 in France). Japanese respondents expressed significantly stronger resistance compared with those in the United Kingdom for GM drought-tolerant crops (*P*<.001) and GM cold weather-tolerant crops (*P*<.001, g=−.25, CI: −0.378 to −0.112). In addition, Japanese respondents expressed significantly stronger resistance compared to those in the United States (*P*<.001, g=−0.32, CI: −0.448 to −0.181) and the United Kingdom (*P*<.001, g=−0.41, CI: −0.542 to −0.274) for GM rapid-grow apples (Table 3, Figure 2).

**Table 3:** Resistance to GM^a^ versus breeding-improved products.^b,c^

Item		Values	Japan	United States	the United Kingdom	France	ANOVA^d^ (F value)	*P*
GM products								
	Salmon that grows twice as fast as traditional salmon								
		Mean (SD^e^)	2.60 (1.186)	2.77 (1.495)	2.80 (1.453)	2.07 (1.173)	26.618	<.001
		Effect size:g(CI)		−0.12 (−0.26 to 0.01)	−0.15 (−0.29 to −0.02)	0.45 (0.31 to 0.58)		
	Killifish whose bodies shine like tropical fish								
		Mean (SD)	2.76 (1.279)	2.81 (1.490)	2.80 (1.481)	2.25 (1.282)	16.090	<.001
		Effect size:g(CI)		−0.04 (−0.17 to 0.10)	−0.03 (−0.16 to 0.10)	0.40 (0.26 to 0.53)		
	Rose with blue-colored blossoms								
		Mean (SD)	3.60 (1.339)	4.03 (1.567)	3.80 (1.555)	3.46 (1.655)	10.982	<.001
		Effect size:g(CI)		−0.29 (−0.43 to −0.16)	−0.13 (−0.27 to 0.00)	0.10 (−0.03 to 0.23)		
	Rice that relieves symptoms of hay fever when continuously consumed								
		Mean (SD)	3.29 (1.250)	3.47 (1.529)	3.50 (1.529)	2.70 (1.358)	28.565	<.001
		Effect size:g(CI)		−0.13 (−0.26 to 0.01)	−0.15 (−0.28 to −0.01)	0.46 (0.32 to -0.59)		
	Crops resistant to certain herbicides or weed killers								
		Mean (SD)	2.521.223	3.31 (1.500)	3.53 (1.459)	2.36 (1.263)	75.926	<.001
		Effect size:g(CI)		−0.58 (−0.71 to −0.44)	−0.76 (−0.89 to −0.62)	0.13 (0.00 to 0.26)		
	Crops resistant to specific harmful pests								
		Mean (SD)	2.68 (1.233)	3.41 (1.515)	3.63 (1.483)	2.64 (1.319)	55.933	<.001
		Effect size:g(CI)		−0.53 (−0.66 to −0.39)	−0.70 (−0.84 to −0.56)	0.03 (−0.10 to 0.16)		
	Crops enriched with specific nutrients such as vitamin C, etc.								
		Mean (SD)	3.18 (1.178)	3.81 (1.486)	3.74 (1.456)	2.98 (1.349)	38.545	<.001
		Effect size:g(CI)		−0.48 (−0.61 to −0.34)	−0.43 (−0.56 to −0.29)	0.16 (0.03 to 0.29)		
	Crops that make efficient use of water and grow in arid or drought-stricken environments								
		Mean (SD)	3.52 (1.194)	3.77 (1.489)	3.92 (1.464)	3.68 (1.622)	5.879	<.001
		Effect size:g(CI)		−0.19 (−0.32 to −0.05)	−0.30 (−0.44 to −0.17)	−0.12 (−0.25 to 0.02)		
	Crops resistant to cold weather and extremely low temperatures								
		Mean (SD)	3.54 (1.228)	3.76 (1.486)	3.87 (1.391)	3.00 (1.392)	33.284	<.001
		Effect size:g(CI)		−0.16 (−0.29 to −0.03)	−0.25 (−0.38 to −0.11)	0.42 (0.29 to 0.55)		
	Apples that ripen faster and can be picked sooner than regular apples								
		Mean (SD)	2.99 (1.227)	3.41 (1.467)	3.54 (1.464)	2.59 (1.314)	41.780	<.001
		Effect size:g(CI)		−0.32 (−0.45 to −0.18)	−0.41 (−0.54 to −0.27)	0.32 (0.18 to 0.45)		
Breeding-improved products								
	Salmon that grows twice as fast as traditional salmon								
		Mean (SD)	3.16 (1.303)	3.47 (1.617)	3.41 (1.553)	2.44 (1.392)	42.752	<.001
		Effect size:g(CI)		−0.21 (−0.35 to −0.08)	−0.18 (−0.31 to −0.04)	0.53 (0.39 to 0.66)		
	Killifish whose bodies shine like tropical fish								
		Mean (SD)	3.26 (1.362)	3.33 (1.610)	3.29 (1.559)	2.52 (1.419)	28.371	<.001
		Effect size:g(CI)		−0.05 (−0.18 to 0.08)	−0.02 (−0.16 to 0.11)	0.53(0.39 to 0.66)		
	Rose with blue-colored blossoms								
		Mean (SD)	3.90 (1.353)	4.28 (1.481)	4.07 (1.526)	3.56 (1.699	16.749	<.001
		Effect size:g(CI)		−0.27 (−0.41 to −0.14)	−0.12 (−0.25 to 0.01)	0.22 (0.08 to 0.35)		
	Rice that relieves symptoms of hay fever when continuously consumed								
		Mean (SD)	3.67 (1.285)	3.94 (1.472)	3.96 (1.475)	2.95 (1.483)	44.944	<.001
		Effect size:g(CI)		−0.20 (−0.33 to −0.06)	−0.21 (−0.34 to −0.08)	0.52 (0.38 to 0.65)		
	Crops resistant to certain herbicides or weed killers								
		Mean (SD)	3.23 (1.329)	3.89 (1.492)	4.07 (1.435)	2.76 (1.451)	75.119	<.001
		Effect size:g(CI)		−0.47 (−0.60 to −0.33)	−0.61 (−0.74 to −0.47)	0.34 (0.21 to 0.47)		
	Crops resistant to specific harmful pests								
		Mean (SD)	3.30 (1.347)	4.01 (1.494)	4.13 (1.442)	2.91 (1.497)	68.251	<.001
		Effect size:g(CI)		−0.51 (−0.64 to −0.37)	−0.60 (−0.73 to −0.46)	0.27 (0.14 to 0.41)		
	Crops enriched with specific nutrients such as vitamin C, etc.								
		Mean (SD)	3.62 (1.247)	4.17 (1.434)	4.14 (1.391)	3.15 (1.515)	49.784	<.001
		Effect size:g(CI)		−0.41 (−0.54 to −0.28)	−0.39 (−0.52 to −0.26)	0.34 (0.21 to 0.47)		
	Crops that make efficient use of water and grow in arid or drought-stricken environments								
		Mean (SD)	3.91 (1.265)	4.26 (1.368)	4.23 (1.393)	3.62 (1.650)	18.882	<.001
		Effect size:g(CI)		−0.27 (−0.40 to −0.14)	−0.24 (−0.37 to −0.11)	0.20 (0.06 to 0.33)		
	Crops resistant to cold weather and extremely low temperatures								
		Mean (SD)	3.87 (1.265)	4.18 (1.405)	4.22 (1.405)	3.16 (1.513)	51.176	<.001
		Effect size:g(CI)		−0.23 (−0.37 to −0.10)	−0.26 (−0.39 to −0.13)	0.51 (0.38 to 0.65)		
	Apples that ripen faster and can be picked sooner than regular apples								
		Mean (SD)	3.49 (1.277)	4.00 (1.473)	4.00 (1.464)	2.90 (1.441)	56.599	<.001
		Effect size:g(CI)		−0.37 (−0.50 to −0.24)	−0.38 (−0.51 to −0.24)	0.43 (0.29 to 0.56)		

^a^GM: genetically modified.

^b^Likert Scale: 1= very strong resistance to 6= no resistance at all.

^c^Mean: average of Likert Scale points.

^d^ANOVA: analysis of variance.

^e^SD: standard deviation.

### Resistance to Breeding-Improved Products

#### Comparison of Demographics

Multiple regression analyses showed significant differences in all questions for gender (*P*<.001); country (*P*<.001) except for breeding-improved pest-resistant crops (*P*=.007) and drought-tolerant crops (*P*=.005); and education for breeding-improved salmon (*P*=.014), shining killifish (*P*=.023), crops that relieve symptoms of hay fever when continuously consumed (*P*<.001), crops resistant to certain herbicides or weed killers (*P*=.003), crops resistant to specific harmful pests (*P*=.003), crops enriched with specific nutrients (*P*<.001), crops that make efficient use of water and grow in arid or drought-stricken environments (*P*<.001), crops resistant to cold weather and extremely low temperatures (*P*=.001), and apples that ripen faster and can be picked sooner than regular apples (*P*=.003) (Table 3).

With *t* tests, females felt stronger resistance for all breeding-improved products in the United Kingdom, and all except a couple of items in Japan and the United States, and some items in France: breeding-improved salmon (*P*<.001 in the United Kingdom, Japan, and the United States); breeding-improved shining killifish (*P*<.001 in the United Kingdom, *P*=.030 in Japan, and *P*=.010 in the United States); breeding-improved blue roses (*P*=.012 in the United Kingdom); breeding-improved hay fever-alleviating rice (*P*=.002 in the United Kingdom, *P*=.001 in Japan, *P*=.001 in the United States, and *P*<.001 in France); breeding-improved herbicide-tolerant crops (*P*=.004 in the United Kingdom, *P*<.001 in Japan, *P*=.015 in the United States, and *P*=.046 in France); breeding-improved pest-resistant crops (*P*=.008 in the United Kingdom, *P*<.001 in Japan, *P*=.007 in the United States, and *P*=.014 in France); breeding-improved nutrient-enriched crops (*P*=.044 in the United Kingdom, and *P*=.002 in Japan); breeding-improved drought-tolerant crops (*P*=.010 in the United Kingdom, *P*=.001 in Japan, and *P*=.022 in the United States); breeding-improved cold weather-tolerant crops (*P*=.009 in the United Kingdom, *P*<.001 in Japan, *P*=.005 in the United States, and *P*=.035 in France); and breeding-improved rapid-grow apples (*P*<.001 in the United Kingdom, Japan, and the United States; and *P*=.021 in France).

Respondents without higher education showed significantly stronger resistance in all items in the United States, in all except one item in the United Kingdom, in some items in Japan, and one item in France: breeding-improved salmon (*P*=.030 in the United Kingdom, and *P*=.005 in the United States); breeding-improved shining killifish (*P*=.007 in the United Kingdom, *P*=.026 in the United States, and *P*=.039 in France); breeding-improved blue roses (*P*=.007 in the United Kingdom, and *P*=.017 in the United States); breeding-improved hay fever-alleviating rice (*P*=.005 in the United Kingdom, *P*=.031 in Japan, and *P*=.002 in the United States); breeding-improved herbicide-tolerant crops (*P*=.001 in the United Kingdom, and *P*=.024 in the United States); breeding-improved pest-resistant crops (*P*=.003 in the United Kingdom, and *P*=.002 in the United States); breeding-improved nutrient-enriched crops (*P*=.013 in Japan, and *P*<.001 in the United States); breeding-improved drought-tolerant crops (*P*=.030 in the United Kingdom, *P*=.015 in Japan, and *P*<.001 in the United States); breeding-improved cold weather-tolerant crops (*P*=.002 in the United Kingdom, *P*=.018 in Japan, and *P*<.001 in the United States); and breeding-improved rapid-grow apples (*P*<.001 in the United Kingdom, *P*=.020 in Japan, and *P*=.002 in the United States).

#### Comparison of Countries

Overall, resistance to breeding-improved products is about 20% weaker than to GM products (Figure 1). Comparisons among countries revealed significant differences. For all breeding-improved items, France showed significantly stronger resistance than the other 3 countries (*P*<.001). Japan showed stronger resistance to breeding-improved salmon (*P*=.009, g=−0.21, CI: −0.347 to −0.081) than the United States. Japan also showed stronger resistance to 7 breeding-improved products than the United States and the United Kingdom: breeding-improved hay fever-alleviating rice (*P*=.025, g=−0.20, CI: −0.331 to −0.064 for the United States, and *P*=.015, g=−0.21, CI: −0.343 to −0.076 for the United Kingdom), herbicide-tolerant crops (*P*<.001, g=−0.47, CI: −0.603 to −0.333 for the United States, and *P*<.001, g=−0.61, CI: −0.740 to −0.469 for the United Kingdom), pest-resistant crops (*P*<.001 for both the United States and the United Kingdom), nutrient-enriched crops (*P*<.001 for both the United States and the United Kingdom), drought-tolerant crops (*P*=.001, −0.27, CI: −0.404 to −0.137 for the United States, and *P*=.005, g=−0.24, CI: −0.374 to −0.108 for the United Kingdom), cold weather-tolerant crops (*P*=.006, g=−0.23, CI: −0.365 to −0.099 for the United States and *P*=.001, g=−0.26, CI: −0.394 to −0.127 for the United Kingdom), and rapid-grow apples (*P*<.001, g=−0.37, CI: −0.504 to −0.236 for the United States, and *P*<.001, g=−0.38, CI: −0.509 to −0.241 for the United Kingdom, Table 3).

### Perception of Genetically Modified Technology and Food

#### Comparison of Demographics

Country showed significant differences for all items, and age in some products in the multiple linear regression analyses. For country: “1. Most consumers are not aware of risks to food safety” (*P*<.001), “2. Most consumers do not understand the risk of GM food” (*P*=.010), “3. If provided with an explanation of genetically modified technology, most consumers would accept GM food” and “4. Most consumers would accept GM food if provided with scientific data supporting its safety” (*P*<.001), “5. Most consumers would accept GM food if they understood that all food carries a certain level of risk” (*P*=.006), “6. Most consumers cannot understand genetically modified technology even if it is explained to them” (*P*<.001), “7. Consumers should try hard to understand scientific information and learn more about the issue” (*P*=.009), and “8. It is annoying to hear the same argument about safety of GM food repeated over and over, even when consumers don’t understand it” (*P*=.023). For age: “6. Most consumers cannot understand genetically modified technology even if it is explained to them” (*P*<.001), “7. Consumers should try hard to understand scientific information and learn more about the issue” (*P*=.003), and “8. It is annoying to hear the same argument about safety of GM food repeated over and over, even when consumers don’t understand it” (*P*=.031). With 1-way ANOVA, Japan did not show any significance for age, but other countries showed significant differences. With the Tukey tests, for “1. Most consumers are not aware of risks to food safety,” only France showed a significant difference between respondents in their 40s and those in their 50s (*P*=.018). For “2. Most consumers do not understand the risk of GM food,” the United Kingdom showed respondents in their 50s significantly agreed compared to those in their 30s (*P*=.012), and France showed respondents in their 50s significantly agreed compared to those in their 60s (*P*=.012).

For “3. If provided with an explanation of genetically modified technology, most consumers would accept GM food,” only respondents in their 60s in the United Kingdom significantly strongly disagreed compared to those in their 50s (*P*<.001). For “4. Most consumers would accept GM food if provided with scientific data supporting its safety,” only UK respondents in their 60s significantly strongly disagreed compared to those in their 50s (*P*=.007). For “5. Most consumers would accept GM food if they understood that all food carries a certain level of risk,” only the United Kingdom showed significance for respondents in their 60s compared to those in their 30s (*P*=.036) and 50s (*P*<.001). For “6. Most consumers cannot understand genetically modified technology even if it is explained to them,” only USA respondents in their 60s significantly strongly agreed compared to those in their 30s (*P*=.043). For “7. Consumers should try hard to understand scientific information and learn more about the issue,” respondents in their 30s significantly strongly disagreed compared to those in their 50s (*P*=.033 in the United Kingdom and *P*=.42 in France). For “8. It is annoying to hear the same argument about safety of GM food repeated over and over, even when consumers don’t understand it,” only France showed significant strongly agree for respondents in their 30s compared to those in their 40s (*P*=.013), and between those in their 40s and 50s (*P*=.003).

#### Comparison of Countries

A comparison among countries showed significance for each item (*P*<.001). Tukey tests showed that most respondents in the 3 participating countries other than Japan were not as aware of food safety, whereas Japanese respondents were significantly strongly aware of food safety (*P*<.001, g=0.50, CI: 0.367-0.637 for the United States; *P*=.003, g=0.23, CI: 0.095-0.361 for the United Kingdom; and *P*<.001, g=0.84, CI: 0.701-0.979 for France). Furthermore, around 90% of respondents in each country agreed that “2. Most consumers do not understand the risk of GM food,” and respondents in France agreed with the statement more than those in Japan (*P*=.017, g=0.23, CI: 0.093-0.359) and the United Kindom (*P*=.017, g=−0.19, CI: −0.330 to −0.057; Table 4).

**Table 4:** Recognition of risk from GM^a^ technology and food.^b-d^

Item	Values	Japan	United States	the United Kingdom	France	ANOVA^e^ (F value)	*P*
1. Most consumers are not aware of risks to food safety.								
	Mean (SD^f^)	2.96 (1.066)	2.39 (1.184)	2.69 (1.287)	2.07 (1.047)	48.112	<.001
	Effect size:g (CI)		0.50 (0.37 to 0.64)	0.23 (0.09 to 0.36)	0.84 (0.70 to 0.98)		
2. Most consumers do not understand the risk of GM food.								
	Mean (SD)	2.27 (0.862)	2.18 (1.141)	2.28 (1.224)	2.06 (1.024)	3.902	.009
	Effect size:g (CI)		0.09 (−0.04 to 0.22)	−0.01 (−0.14 to 0.13)	0.23 (0.09 to 0.36)		
3. If provided with an explanation of genetically modified technology, most consumers would accept GM food.								
	Mean (SD)	3.39 (1.081)	3.12 (1.225)	3.19 (1.234)	2.93 (1.321)	10.709	<.001
	Effect size:g (CI)		0.23 (0.10 to 0.36)	0.17 (0.04 to 0.30)	0.38 (0.25 to 0.52)		
4. Most consumers would accept GM food if provided with scientific data supporting its safety.								
	Mean (SD)	3.26 (1.073)	2.93 (1.236)	3.03 (1.188)	2.82 (1.288)	10.546	<.001
	Effect size:g (CI)		0.28 (0.15 to 0.42)	0.20 (0.07 to 0.33)	0.37 (0.23 to 0.50)		
5. Most consumers would accept GM food if they understood that all food carries a certain level of risk.								
	Mean (SD)	3.42 (1.059)	3.16 (1.258)	3.22 (1.199)	3.16 (1.415)	4.443	.004
	Effect size:g (CI)		0.22 (0.09 to 0.35)	0.18 (0.05 to 0.31)	0.21 (0.08 to 0.35)		
6. Most consumers cannot understand genetically modified technology even if it is explained to them.								
	Mean (SD)	3.18 (1.027)	2.97 (1.299)	2.98 (1.226)	2.80 (1.304)	6.948	<.001
	Effect size:g (CI)		0.17 (0.04 to 0.31)	0.18 (0.05 to 0.31)	0.32 (0.19 to 0.45)		
7. Consumers should try hard to understand scientific information and learn more about the issue.								
	Mean (SD)	2.68 (0.898)	2.45 (1.159)	2.66 (1.125)	2.86 (1.370)	8.659	<.001
	Effect size:g (CI)		0.22 (0.08 to 0.35)	0.02 (−0.12 to 0.15)	−0.16(−0.29 to −0.03)		
8. It is annoying to hear the same argument about safety of GM food repeated over and over, even when consumers don’t understand it.								
	Mean (SD)	3.12 (1.047)	3.22 (1.326)	3.34 (1.198)	2.87 (1.409)	11.050	<.001
	Effect size:g (CI)		−0.08 (−0.22 to 0.05)	−0.20 (−0.33 to −0.06)	0.21 (0.08 to 0.34)		

^a^GM: genetically modified.

^b^“Consumers” in this paper means nonexperts.

^c^Likert Scale: 1= strongly agree → 6= strongly disagree.

^d^Mean: average of Likert Scale points.

^e^ANOVA: analysis of variance.

^f^SD: standard deviation.

France significantly agreed that most consumers would accept GM food “if provided with an explanation of GM technology," “if provided with scientific data supporting its safety,” and “if most consumers understand that any food carries a level of risk.” However, Japanese respondents showed significant disagreement with these statements: “3. If provided with an explanation of genetically modified technology, most consumers would accept GM food” for the United States (*P*=.007, g=0.23, CI: 0.096-0.363) and for France (*P*<.001, g=0.38, CI: 0.250-0.518); “4. Most consumers would accept GM food if provided with scientific data supporting its safety” for the United States (*P*<.001, g=0.28, CI: 0.151-0.418), the United Kingdom (*P*=.026, g=0.20, CI: 0.068-0.334), and France (*P*<.001, g=0.37, CI: 0.233-0.501); and “6. Most consumers would accept GM food if they understood that all food carries a certain level of risk” for the United States (*P*=.012, g=0.22, CI: 0.088-0.355) and France (*P*=.009, g=0.21, CI: 0.079-0.346). Furthermore, 58% of respondents in Japan agreed that “6. Most consumers cannot understand GM technology, even if it is explained to them,” whereas about 70% of respondents in the other 3 countries showed agreement; significantly fewer respondents in Japan think that GM technology is understandable than in France (*P*<.001, g=0.32, CI: 0.187-0.454; Table 4).

For the statement “7. Most consumers should try hard to understand scientific information and learn more about the issue,” a significant difference was observed among countries; USA respondents showed significance in agreement compared with those in Japan (*P*=.022, g=0.22, CI: 0.082-0.348) and France (*P*<.001, g=−0.16, CI: −0.292 to −0.025; Table 4). For the statement, “8. It is annoying to hear the same argument about safety of GM food repeated over and over, even when consumers don’t understand it,” the respondents who agreed were 67%, 70%, 61%, and 59% in Japan, France, the United States, and the United Kingdom, respectively. With a Tukey test, a significant difference among the countries was shown; French respondents agreed significantly more with this statement than respondents in Japan (*P*=.012, g=0.21, CI: 0.077-0.343), the United States (*P*<.001, g=−0.262, CI: −0.398 to −0.125), and the United Kingdom (*P*<.001, g=−0.366, CI: −0.502 to −0.228; Table 4).

### Willingness to Pay to Measure Resistance to Genetically Modified Food

**Figure 3:**
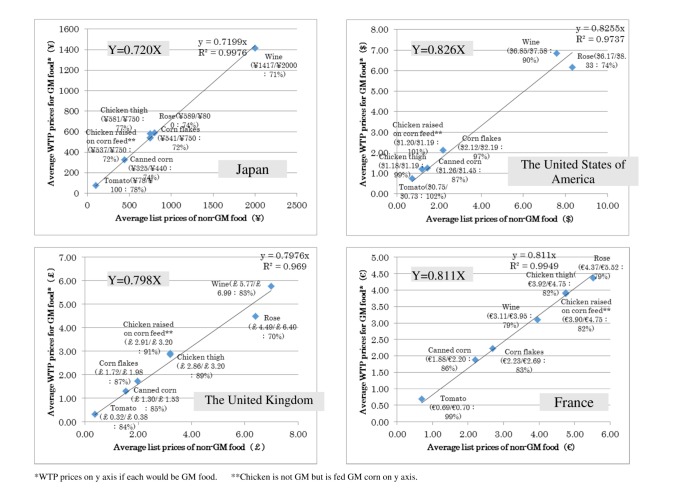
Comparison of WTP for GM and non-GM food. GM: genetically modified, WTP: willingness to pay.

Participants who answered that they intended to purchase GM products were asked to indicate their WTP for GM food. Japanese consumers were willing to accept about a 30% discount for GM food compared to the average market-list price for comparable non-GM food, whereas respondents in the other 3 countries would accept a discount of approximately 20% for GM food (Figure 3).

## Discussion

### Principal Findings

This study attempted to illustrate Japanese perceptions of GM food in comparison with 3 other countries.

More than expected in H1, demographic factors of gender, age, and education overall seem to somewhat influence consumers’ perceptions; females, people in their 50s and older, and those with less education tended to show strong resistance to GM food in this study. These results confirmed previous studies that some demographic items were related to consumer perceptions [14,35]. However, France was not entirely in line with these results. Empirically, people who have a child or children may be more sensitive to perceived risks so we could surmise that they may exhibit resistance to GM food. Contrary to this empirical speculation, however, people with children did not show significant resistance to GM food in this study. In this and previous studies, each nation’s experiences and social factors contribute to the evolution of consumer perceptions [8-13,29,30,35,36]. This study showed that these demographic factors are still influencing, but not determinants of, consumer perceptions.

Even though GM food has no documented health risks thus far, affect/emotional reactions to perceived risk appear to be stronger than cognitive understanding [35]. In this case, although educational approaches that present information consumers need to know—as opposed to what they want to know—have not been shown to promote perception and attitude change, ongoing education that specifically addresses consumers’ concerns may reduce their fear and help them understand GM technology [3,14,29,37]. Effective educational materials should be examined in future research.

One aim of this research was to determine how fear of health hazards may disturb the intent to understand and accept GM food as shown in H2. Previous studies discussed that consumers often recognize that food purchased from the market is largely safe to eat [38], and food choices frequently reflect compromises in consumers’ life style rather than their preferences [29]. In this study, however, consumers in all participating countries showed a degree of fear of health hazards from food contamination. Especially, Japanese respondents expressed the strongest fear and/or sensitivity to health hazards from food compared to those in other countries (Table 2). Consumers in Japan and France seemed to recognize GM food as something that poses a health risk, and showed stronger resistance than the United States and the United Kingdom.

Based on the results of the association between the perception that GM food poses a health hazard and the perceptions related to GM technology and food, even consumers who believe that GM food poses a health hazard desire scientific data to support its safety; they appear not to have enough data to confirm GM food safety. Therefore, US consumers are aware that they also need to study scientific information about products they consume. Contrary to the United States, however, consumers in France who thought GM food poses a health hazard expressed annoyance at repeatedly having to hear the same argument about the safety of GM food. The countries with high levels of uncertainty avoidance, such as Japan and France, may be influenced in their resistance to GM food and seek solid answers about GM food [25,26]. Under these circumstances, having more information may not lead to a solution that resolves the uncertainty presented by GM food; consumers in France may become irritated by explanations of GM food and still remain uncertain.

When the term “GM” was replaced with the term “breeding improved” for the same products, as stated in H3, consumer resistance was reduced (Table 3). The results of this study suggest that “GM” may already have a negative connotation in consumers’ minds, especially in Japan and France, which have the strongest resistance to GM food. In addition, it was considered that people may not have enough information about GM food to construct their attitudes and may be more influenced emotionally by perceived risk. This is in line with previous reports showing that affect influences perceived risk [8,35].

Although this study did not formulate a hypothesis for consumers’ acceptance of specific GM food items, we additionally found that all 4 countries showed some resistance to GM products, but there was variance among them. GM organisms, such as GM salmon, evoked stronger resistance than GM crops in this study, a finding that was also reported by previous studies [3,15,29]. GM products with advantages primarily for product producers, such as GM herbicide-tolerant and pest-resistant crops, also showed strong resistance. However, GM products that have direct advantages for consumers and/or have advantages under certain daily and/or environmental conditions appeared to evoke relatively less resistance.

For instance, hay fever is such a common seasonal symptom in Japan that it is called “the national disease” due to the large number of people who suffer from it. Another example from France is the severe drought in 2003 that led to extreme aridity and the water restrictions that were put in place in 2011; among French consumers, resistance to drought-tolerant GM food was relatively weaker in our study, although a link to consumers’ experiences of the environmental and social events of 2003 and 2011 was not examined in this study (Figure 3). Previous studies have argued that a mix of factors, including social and psychological, and the risks and benefits of each food, may influence risk perceptions [9-13,29,30], affect [35], moral convictions, fairness [8], and attitudes regarding the benefits and risks of GM food [36]. Although a direct relationship between the level of resistance and specific natural and industrial disasters, as well as food-related incidents such as food poisoning, were not investigated in this study, we can still speculate that the circumstance and experiences of each country can affect the level of resistance to GM food.

Although Hoban (1997) [2] and Hoban (1999) [15] found that consumers in Japan and the United States have shown relatively weak reactions to GM food and seem to have no great concern and/or objection to GM food becoming commercially available, the results of this study indicated otherwise, confirming H4. The results from WTP also support this finding. Japanese respondents who do not mind purchasing GM food showed the strongest resistance to GM food in the WTP questions compared to the other countries. Contrary to findings in a previous study that only USA consumers would accept GM food if they could purchase it at a discounted price [39], this study found that consumers in the United States, France, and the United Kingdom who did not mind purchasing GM food showed the same level of discounted price in the WTP questions. The stronger resistance shown by consumers willing to purchase GM food in Japan compared to the United States, France, and the United Kingdom indicates that there may be much stronger unexpressed resistance among Japanese consumers who are not willing to purchase GM food.

Understanding the personal benefits of GM food, and the ability to purchase it at more than 30% off the list price for comparable non-GM food may be effective communication cues for Japanese consumers, as would be about a 20% discount for consumers in the other participating countries. Although the impact of such communication on consumers may be short lived, it can be used to start discussions about GM food and its safety among consumers. In this regard, it should be stated that Japanese nonverbal reactions are sometimes stronger than superficially expressed; people tend to be reluctant to freely express individual opinions or attitudes because they value social balance and prefer to accommodate to the situational context [27,28]. To account for this cultural difference, it is important that future research on Japanese consumer perceptions develop a tool to detect and measure discrepancies that may exist between a person’s verbalized response and their emotional reaction.

Contrary to Japanese consumers’ negative perceptions of GM food, they appeared to accept GM technology slightly more than those in the other countries, which was not in line with H5, while still rejecting its application to GM food. Japanese and French consumers may be unlikely to accept GM food even if shown scientific evidence of its safety, provided with understandable explanations of GM food, and helped to understand that consuming any food carries a risk, even though they seem to desire safety. This lack of information may have several effects: it may increase their uncertainty [21]; it may strengthen the belief that GM food causes health hazards to create certainty [9]; or it may tighten their hold on preexisting attitudes [23]. Furthermore, Japanese and French consumers’ high level of uncertainty avoidance may also influence their perception not to accept GM food [26]. Under these circumstances, continuously providing scientific and other necessary information may, in fact, lead to greater feelings of distrust and may disturb Japanese consumers’ proactive thinking about GM food [6,7].

Although Japanese consumers showed slightly less resistance toward GM technology, there is a gap between welcoming the advanced technology and accepting its use in food production. As Siegrist [35] stated, perception of GM technology seemed to depend on its application. It is still unclear whether perception and acceptance of GM technology by Japanese consumers depends on the type of GM technology application. The mechanisms between their perceptions of GM food and their experiences should be examined in a future study.

### Conclusion

As the results of this study show, every participating country showed a degree of resistance to GM food; however, France and Japan had overall stronger resistance than the United States and the United Kingdom. It appeared that each country’s experiences may be related to its consumers’ acceptance of GM food. In fact, the term, “GM food” itself seemed to already carry a negative connotation. The belief that GM food poses health hazards is likely to be associated with the perception of GM food, which, in turn, appears to be related to their cultural predispositions toward uncertainty avoidance. Consumers in each country would like the assurance of scientific data proving that GM food is safe, but as long as such assurance is not provided consumers in each country may rely on less information to create their perceptions and attitudes, be less likely to seek out more information regarding GM food, and may not accept GM food.

To motivate and influence processing of information about GM food, it may be more effective to use the ELM peripheral route, employing communication cues that emphasize benefits to consumers, including setting discount prices, constantly providing information to overcome each country’s experiences as well as ensure the safety of GM food. Basically, cultural differences among the participating countries did not appear to strongly influence acceptance or nonacceptance of GM food. Therefore, some measurements developed in Western cultures would be adaptable to the Japanese context. However, we must keep in mind that Japanese cultural traits that place a high importance on social balance and harmony may demotivate consumers to express their true opinion. This cultural predisposition should be carefully considered and measured in future studies.

### Limitations

A limitation of our study design is that it excluded people who are not familiar with the Internet and do not use a computer. However, taking into consideration rising rates of computer and Internet use, the increasing acceptance of Web-based academic studies, and the quality control implemented by Macromill and Tokyo to prevent invalid responses, our model for conducting Web-based studies remains an effective way to collect international data.

Furthermore, the recruitment rate for the Web-based survey employed in this study was low, which may bias the results. We were only able to communicate with our international respondents via email to remind them to complete the questionnaire. Even with this limitation, however, we were able to obtain at least 400 completed questionnaires from each of the 4 countries surveyed, a number sufficient for meaningful statistical analyses and to yield important information about Japanese consumer characteristics and how they compare to those of the other 3 nations.

We chose 3 countries to compare: the United States, the United Kingdom, and France. Selecting these countries might bias this research. However, comparison with these countries provided new perspectives and insights about GM food for Japanese consumers and those in the other nations selected. In the future, we would like to conduct studies comparing Japanese perceptions with other countries than those in this study.

This study focused on health hazards as a reason for resistance, which is one of the main trends of discussion regarding GM food risks. Mechanisms to reduce this fear were not examined in this study. Further studies should be conducted to evaluate the reaction mechanisms of Japanese consumers to other risk-relevant concepts, such as environmental conditions, consumers’ rights, source characteristics, and the benefits of GM food. However, it remains meaningful to observe the unique characteristics of Japanese consumer perceptions toward risk and to provide new perspectives for the participating countries.

In hindsight, several questions in the survey were double-barreled. Although the aim of this study was not affected, we need to revise these questions in future studies. As for the cultural influences alluded to in this study, we need to conduct future studies that focus solely on such cultural differences.

## Abbreviations

CFA: confirmatory factor analysis
EFA: exploratory factor analysis
ELM: elaboration likelihood model
GM: genetically modified
WTP: willingness to pay
